# Thermal Properties of Eco-Friendly Earthen Materials Stabilized with Bio-Based Polymers: Experimental Data and Modeling Procedure for Improving Mix-Design

**DOI:** 10.3390/ma17051035

**Published:** 2024-02-23

**Authors:** Marta Cappai, Rizwan Shoukat, Luca Pilia, Roberto Ricciu, Daniele Lai, Gianluca Marongiu, Giorgio Pia

**Affiliations:** 1 Dipartimento di Ingegneria Meccanica, Chimica e dei Materiali, Università degli Studi di Cagliari, Via Marengo 2, 09123 Cagliari, Italy; marta.cappai@unica.it (M.C.); rizwan.shoukat@unica.it (R.S.); daniele.lai@unica.it (D.L.); gianluca.marongiu@unica.it (G.M.); 2 Materialia Association, 09037 San Gavino Monreale, Italy; 3 Dipartimento di Ingegneria Civile, Ambientale e Architettura, Università degli Studi di Cagliari, Via Marengo 2, 09123 Cagliari, Italy; ricciu@unica.it

**Keywords:** bio-based polymer, earth-based materials, eco-friendly materials, mix-design, modeling, thermal properties

## Abstract

The fight against climate change has delineated new objectives, among which one of the most crucial is the replacement of high-energy-intensity materials in the construction sector with more sustainable and thermally efficient alternatives to reduce indirect emissions. Consequently, the thermal properties of materials assume fundamental importance. In this regard, the large-scale use of earth represents a promising option, not only due to its widespread availability but especially for its minimal embodied energy. However, to enhance its durability, it is necessary to stabilize the mixtures of raw materials. This study analyzes experimental systems based on earth stabilized with bio-based polymers to evaluate their thermal properties and how these vary depending on the selected mix-design. The experimental measurements showed thermal properties comparable to conventional materials. As expected, thermal conductivity increases when porosity decreases. The minimum value is equal to 0.216 W/m·K vs. a porosity of 43.5%, while the maximum is 0.507 W/m·K vs. a porosity of 33.2%. However, the data obtained for individual systems may vary depending on the topological characteristics, which were analyzed through a model for granular materials. The modeling suggests correlations between microstructures and thermal behaviour, which can be useful to develop tools for the mix-design procedure.

## 1. Introduction

Since 2015, the goals of the 2030 agenda have permeated everyday life, calling on each of us to play our part in countering catastrophic climate change in favour of sustainable development. The entire world is called upon to be accountable for this change and, in this regard, the construction sector must take a leading role due to its high CO_2_ emissions and energy consumption.

From 2015 to 2021, direct emissions from construction operations increased by an average of nearly 1% annually [1]. In 2022, there was a change in trend with a decrease in them. However, for the same year (2022), indirect emissions from the production of electricity and heat used in buildings increased by about 1.4%. Consequently, direct CO_2_ emissions from buildings decreased to 3 Gt in 2022, while indirect CO_2_ emissions increased to nearly 6.8 Gt [1].

This is directly due to modern lifestyles and the indoor environmental comfort required.

In addition, it should be considered that the materials used in building construction (concrete, steel, aluminium, glass, and brick) contribute to approximately 9% of the total energy-related CO_2_ emissions [2,3]. It is estimated that the use of raw materials will double by 2060. Therefore, it is evident that the efforts made so far are not sufficient to achieve decarbonization by 2050 [1].

Among building materials, the most impactful is Portland cement, which is responsible for about 7% of the world’s and 4% of the EU’s CO_2_ emissions [4].

In particular, during its production, the calcination process plays a key role in terms of environmental pollution by generating approximately 50–60% of CO_2_ during the thermal decomposition reaction of limestone [5,6]. Additionally, the combustion of fossil fuels for oven operation contributes to about 40% of carbon dioxide emissions [7], with an additional 10% of CO_2_ attributed to indirect emissions related to energy consumption during cement and raw material grinding.

According to the EU climate policy, the cement industry is required to reduce its CO_2_ emissions by about 30% by 2030, with the goal of achieving zero emissions by 2050 [8].

As EU plants are already operating at near-optimal efficiency, the industry seems to be directing its attention toward storage and utilization technologies for carbon capture. Meanwhile, breakthroughs in alternative materials are still being explored as part of efforts to further reduce emissions [4].

In this context, the transition to sustainable building practices is crucial. This path can be pursued both through the use of sustainable materials, ensuring low emissions during their production and service life, and through the use of thermally efficient materials, allowing for high indoor environmental comfort without necessarily resorting to large amounts of energy for heating and cooling.

Many efforts are being made to obtain sustainable binders, such as those based on sulfoaluminate cements or geopolymers [9,10,11,12].

Despite some of these materials being developed as early as the 1940s and 1950s, certain characteristics make their widespread adoption difficult. Currently, the literature on these materials is expanding significantly, but many aspects still require further investigation and improvement. In the case of sulfoaluminate cements, the disadvantages are represented by their overly short setting time [13], low PH values [14], expansion phenomena [15], and high cost [16]. On the other hand, for geopolymers, the drawbacks could be their large shrinkage, high brittleness [17], and mechanical properties, which decrease as a function of time [12,18]. Additionally, the limited familiarity among designers with these types of materials, and their often higher cost compared to Portland cement, restrict their use [11].

Reducing the use of these materials represents an important step forward towards greater sustainability in the construction sector. From this perspective, earth-based materials offer a viable alternative for various applications, such as residential architecture or internal finishing works (plasters) [19,20,21,22,23].

This material, used since ancient times, represents an energy-efficient resource with almost zero embodied energy—less than 0.5 MJ/kg (7.8 MJ/kg for Portland cement) [24,25,26,27,28]. This significant disparity underscores the potential of earthen construction to mitigate the overall environmental footprint of buildings.

Although earth-based systems cannot be considered as insulating materials, their thermal conductivity, which varies from 0.17 to 1.10 W/m·K (as a function of density and mineralogical composition), is similar to other materials commonly used in the construction field, like lime (about from 0.3 to 0.8 W/m·K), cement (about from 0.4 to 1.4 W/m·K), and ceramic bricks (about from 0.4 to 1.5 W/m·K) [29,30,31,32,33,34].

In addition, earthen construction demonstrates thermal efficiency and high thermal comfort standards, especially in climates that have significant temperature variations between day and night [35,36]. Thermal behaviour, due to the high thermal inertia, can also be exploited in cold environments by providing a mass wall within an insulating envelope so that it can store and retain heat within the building and release it slowly during colder hours [36,37,38,39,40]. This aspect could bring many benefits in trying to decrease indirect emissions from the construction sector.

Despite earth-based materials having reduced design flexibility and low mechanical strength (structure vulnerability, earthquakes), they are still compatible with residential architecture applications, especially when paired with other materials. However, an aspect not to be underestimated, from a Materials Science point of view, includes the high variability and heterogeneity of the material, as well as its pronounced sensitivity to water, which can modify its performance, thereby compromising its durability [41,42,43,44]. Moisture can severely impact the mechanical and thermal performance. Research indicates that the presence of moisture can lead to a reduction in compressive strength and an increase in thermal conductivity [45,46]. These factors can have adverse effects on the efficiency and durability of earthen buildings, potentially compromising their sustainability benefits. To address this, researchers have consistently sought to enhance the material’s performance through stabilization methods.

Since ancient times, in many countries around the world, there has been a tendency to use stabilizers of animal and plant origin, dictated by local availability and building traditions [47,48,49,50,51,52]. Today, this stabilization is often obtained by using lime, cements, bitumen, and synthetic polymers [21,48,53,54,55,56,57,58]; however, this tends to undermine the improvements in sustainability offered by earthen materials.

For example, the addition of Portland cement enhances mechanical performance. However, the Global Warming Potential (GWP) associated with unstabilized earth is approximately 23 g. In contrast, for the same quantity of earth stabilized with 5–10% of Portland cement, the GWP reaches about 64–106 g, respectively (for comparison, regular concrete has a GPW of about 130 g) [48].

Regarding synthetic polymers, research has largely stemmed from soil stabilization in the fields of civil engineering and geotechnics. However, as is often the case in earthen construction, this knowledge has also been applied to the realm of earth construction. Due to factors such as cost, chain length, and the possibility of the process not reinitiating once completed, preference has mostly been given to resins obtained through polycondensation. Various types include resorcinol–formaldehyde resins, phenolic resins, furan resins, polyacrylates, and polyurethanes [56,57,58,59,60]. Nevertheless, urea–formaldehyde resins have been the most commonly utilized due to their lower cost [58,59]. One of the drawbacks of this stabilization method is the gradual development of brittleness in the material over time, attributed to resin aging, the recognized toxicity of these compounds, and their fossil-based nature [58,60,61].

Therefore, the necessity for an environmentally friendly, sustainable, and effective approach to enhance earth properties remains a continual challenge for researchers and manufacturers. The utilization of biopolymers can offer an effective solution to improve earth materials’ properties and durability. Indeed, this material class offers a solid basis for conducting further research and fostering the practical application of such materials [62,63,64,65,66].

It is possible to distinguish two macro-categories within the class of biopolymers. These can be natural polymers, occurring naturally, or bio-based polymers, synthesized artificially from renewable natural resources [67].

The incorporation of natural polymers for stabilizing earth has long been a practice embedded in construction traditions. The extensive research conducted over the past two decades has consistently demonstrated the capacity of these materials to enhance the mechanical properties, permeability, and water resistance of earth [66,68,69,70,71,72,73,74].

However, their durability over time could be affected not only by environmental agents but also by their biological vulnerability due to their biodegradable nature (e.g., durability concerning parasite attack and the growth of molds and fungi) [70,75,76]. Additionally, the extreme variety of methodologies used in the production of natural polymers, the variability in testing procedures, and the limited availability of some of them still make it difficult to systematically characterize them to provide stable, standardized, and readily available products for the construction sector [70].

A compromise between synthetic polymers and natural polymers is represented by bio-based polymers. This particular class of biopolymers is synthesized from renewable resources and, in the construction materials sector, they find particular applications in paints and coatings, serving as binders and film-formers [77,78,79,80,81,82,83,84]. However, they have been underexplored in terms of enhancing the performance of earthen materials.

The combination of granular materials constituting the earth and stabilizers represents a delicate step. The resulting properties of the final product, especially the thermal conductivity, are influenced by quantitative and phenomenological factors. In this context, various modeling procedures exist in the literature to enhance our understanding of the thermal behaviour of the resulting materials [85,86,87,88].

However, it is crucial to assess the reliability of these models for the specific material at hand. In this sense, fractal geometry offers some versatile alternatives that seem particularly suitable for replicating the structures of the materials studied in this work [89,90].

## 2. Materials and Methods

The materials used for experimentation were clay (CY) and sand (SD). The CY came from a quarry in Lozzolo, Vercelli (Italy). The SD, on the other hand, came from the Abbazia di Fossanova quarry, Latina (Italy). The two materials were characterized mineralogically, by using a Rigaku^®^ MiniFlex II diffractometer, and granumetrically by wet granulometry according to UNI EN ISO 17892–4:2017 [91].

The two bio-based polymers used in the study were identified as S-BAR and D-UAR. S-BAR is a short oil alkyd emulsion with a bio-based index >95% [77,84] and D-UAR is a urethane–alkyd dispersion with a bio-based index >43% [80]. Some properties of the two bio-based polymers are shown in Table 1.

CY and SD were dry-mixed in a weight percentage composition of 36% and 64%. The proportion used was chosen based on preliminary tests (Appendix A). After the completion of the dry mixing of CY and SD, the slurries were prepared.

Samples were created using the dry mixture of SD and CY, organized into three groups and six sample types. Group 1 involved mixing CY and SD with distilled water, added at 30% (R-30) and 40% (R-40) by weight. Groups 2 and 3, on the other hand, were made using S-BAR and D-UAR in the mixture. The amounts of S-BAR and D-UAR were determined through preliminary tests to assess the consistency of the material (Appendix A). The S series (group 2) was formulated with 5% by weight of S-BAR, and the D series (group 3) with 20% by weight of D-UAR. For both groups, the water content required to obtain a liquid phase (bio-based polymer and water) of 30% and 40% was then added.

The mixtures were placed in molds with dimensions of 105 × 105 × 20 mm. A total of 9 samples were prepared for every mix-design. After 4 days, the molds were removed and the samples were dried under laboratory conditions (23 °C ± 2 °C) for 4 weeks.

Porosimetric measurements, for obtaining open porosity values, were conducted on samples of approximately 1.5 cm^2^ using a Micromeritics^®^ Autopore IV 9500 Porosimeter with Mercury Intrusion Porosimetry (MIP), operating up to 2200 bars with an equilibration time of 10 s. The measurement was repeated three times on each sample.

Thermal conductivity tests were performed according to ISO 8301 [92] using TAURUS TCA 300 equipment and elaborated using software Lambda basic v.1.13.14.0 version.

For each sample, the test was repeated three times by changing the temperature of the cold plate (−5, 0, +5 °C) and the hot plate (15, 20, 25 °C), but keeping the temperature difference between the plates constant (20 °C). The force of the hydraulic piston pushing the hot plate onto the upper face of the sample was equal to 272 N.

## 3. Traditional and Fractal Modeling Approach

The models used in this study represent a reference point in the specialized literature for calculating thermal conductivity (*k*) and effective thermal conductivity (*k_eff_*; *k_eff_* = *k*/*k_s_*) in porous materials. These models utilize values of relative density (φ) or pore fraction (*ε*) along with the conductivity of the solid and the fluid phases (*k_s_* and *k_f_*, respectively). In particular, the considered models are as follows:

- Parallel and series models [85].

(1) $$k_{parallel}=\varphi k_{s}+\left(1-\varphi \right)k_{f}$$

(2) $$k_{series}=\frac{1}{\frac{\left(1-\varphi \right)}{k_{f}}+\frac{\varphi }{k_{s}}}$$

- Hashin and Shtrikman bounds—Maxwell–Eucken (ME: ME1, ME2) [86].

(3) $$k_{ME1}=k_{f}\;\frac{2k_{f}+k_{s}-2\left(k_{f}-k_{s}\right)\varphi }{2k_{f}+k_{s}+\left(k_{f}+k_{s}\right)\varphi }$$

(4) $$k_{ME2}=k_{s}\;\frac{2k_{s}+k_{f}-\left(k_{s}-k_{f}\right)\left(1-\varphi \right)}{2k_{s}+k_{f}+\left(k_{s}-k_{f}\right)\left(1-\varphi \right)}$$

- The Effective Medium Theory (EMT) equation, reported in Carson et al. [87].

(5) $$k_{EMT}=\frac{\left[3\varphi -1\right]k_{s}+\left[3\left(1-\varphi \right)-1\right]k_{f}+\sqrt{{\left\{\left[3\varphi -1\right]k_{s}+\left[3\left(1-\varphi \right)-1\right]k_{f}\right\}}^{2}+8k_{f}k_{s}}}{4}$$

**Figure 1:**
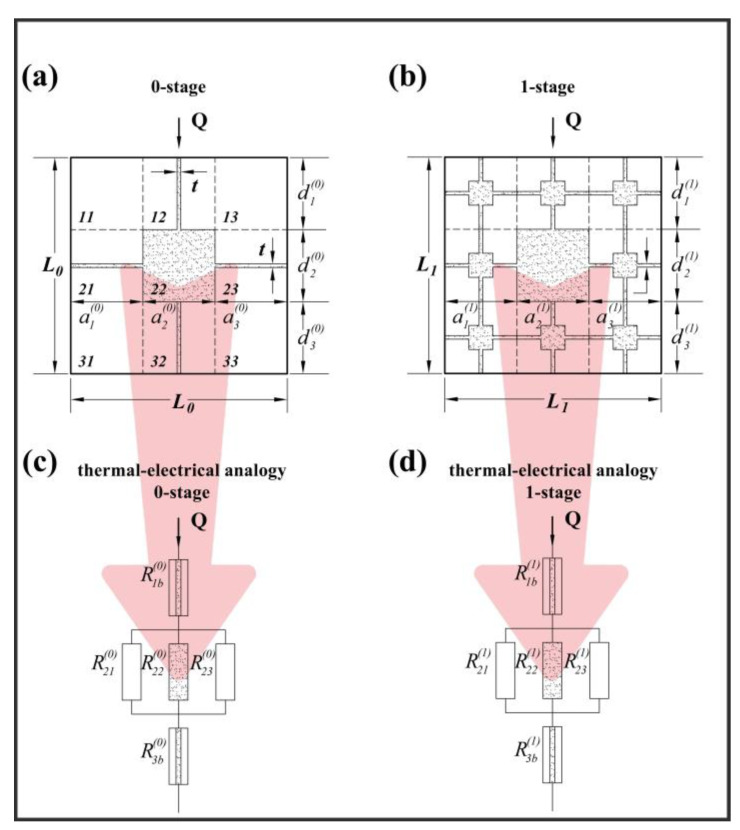
Fractal model scheme and equivalent thermal–electrical pattern for predicting *k*. (**a**) thermal conductivity model for 0-stage and (**b**) for 1-stage; (**c**) thermal-electrical analogy for a 0-stage and (**d**) 1-stage. As reported in Ma et al. and Cappai et al. [88,89], Q is the heat flow rate; L is the length scale, or side length of the Sierpinski carpet; a and d are sub-square lengths; *t* is the barrier thickness; and *R* is the resistance into the thermal electrical analogy pattern.

In addition to these, a model based on fractal geometry, proposed by Ma et al., was selected for studying thermal conductivity in granular porous materials [89,93]. Starting from the assumption that porous materials exhibit fractal characteristics and recursive structures [90], the analytical procedure proposed by Ma et al. utilizes the Sierpinski carpet and its different compositions, such as the geometrical base unit and the scheme for the thermo-electrical analogy technique (Figure 1) [88,89].

The material’s structure is constituted by two distinct parts: the first is composed of non-touching dispersed particles distributed in random way, while the second is marked by a series of touching particles which are arranged symmetrically and are characterized by thermal resistance.

Analytical expressions for this model are represented by the following:

(6) $$k_{e,sc}^{+\left(n\right)}=\frac{3k_{e,sc}^{+\left(n-1\right)}}{2/\left[t^{+}\beta^{\left(n\right)}+\left(1-t^{+}\right)\right]+1/\left(2/3+\beta^{\left(n\right)}/3\right)}$$

(7) $$k_{e}^{+}=\frac{A_{nt}}{A}\left[\left(1-\sqrt{1-\varepsilon }\right)+\frac{\sqrt{1-\varepsilon }}{1+\left(1/\beta -1\right)\sqrt{1-\varepsilon }}\right]+\left(1-\frac{A_{nt}}{A}\right)k_{e,sc}^{+\left(n\right)}$$

where *k^+(n)^_e,sc_* is the dimensionless effective thermal conductivity for an *n*-stage carpet; *t^+^* is dimensionless width of barrier, defined by *t+* = *t*/*Ls* (Figure 1); *k_e_^+^* is the dimensionless effective thermal conductivity; *A* is the total area of representative cross section and *A_nt_* is the equivalent area of a cross section with the same porosity as the non-touching particles (m^2^); and *β* is the ratio of the thermal conductivity of the solid and thermal conductivity of the matrix. In this study, the value of t is fixed to 0.001, as in reference [89].

All terms considered in the analytical formula have a physical meaning. Indeed, the thermal conductivity is expressed as a function of the *ε*, the *A_nt_*/*A*, the ratio between the thermal conductivity of the solid phase, and the thermal conductivity of the matrix [89].

## 4. Results and Discussion

The mineralogical composition of CY, obtained by XRD, consists of 42% minerals belonging to the illitic–kaolinitic group, 38% quartz, 6% potassium feldspar, and 5% sodium feldspar. SD, on the other hand, is composed of 94% quartz.

According to the Wentworth class [94] and the granulometric analysis, the mixture made from CY + SD consists of 65.7% sand (divided in coarse, 0.023%; medium, 2.45%; fine, 52.39%; and very fine, 10.82%) and 34.3% silt and clay.

The values of the average density and average porosity of samples are presented in Table 2. Systems stabilized with bio-based polymers have similar values of density with respect to the as-received samples. Mercury intrusion tests exhibit a large open porosity, which is between 33.4% (D-30) and 43.1% (R-40). The variations are related to the mix design; in particular, when the quantity of the water decreases, the porosity is lower, and when the quantity of the bio-based polymers increases, the porosity decreases.

Thermal conductivity (*k_exp_*) values were measured for the considered specimens (Table 2). This property is influenced by different aspects related to the characteristics of materials, such as the thermal conductivity of solid phases, the porosity, and the consequent thermal conductivity of liquid or gas content, as well as the phenomenological and topological features of their structures (particle shapes, system tortuosity, etc.), mix-design (granulometry, water, and bio-based polymer quantities), and/or fabrication methods (sample compaction, materials application, drying conditions, temperature variations, etc.).

**Figure 2:**
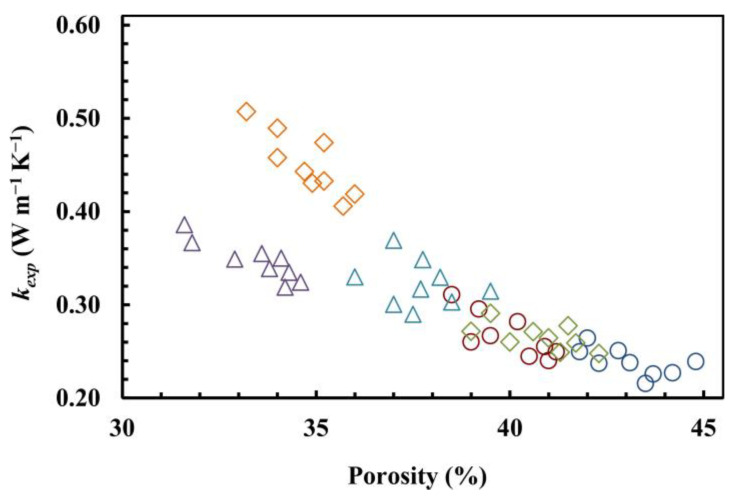
The thermal conductivity (*k_exp_*) as a function of porosity for R-30 (**○**), R-40 (**○**), S-30 (**◊**), S-40 (**◊**), D-30 (**△**), and D-40 (**△**) systems.

As expected, for the same system, the thermal conductivity increases when the porosity decreases (Figure 2). This is more evident for series R and S than series D. Indeed, the minimum value of *k_exp_* recorded for R is equal to 0.216 W/m·K vs. a porosity of 43.5%, while the maximum of *k_exp_* is 0.311 W/m·K vs. a porosity of 38.5%; in the same way, for S, the minimum is 0.248 W/m·K vs. a porosity of 42.5%, while the maximum of *k_exp_* is 0.507 W/m·K vs. a porosity of 33.2%; and, for D, the minimum is 0.290 W/m·K vs. a porosity of 37.5%, while the maximum of *k_exp_* is 0.386 W/m·K vs. a porosity of 31.6%.

**Figure 3:**
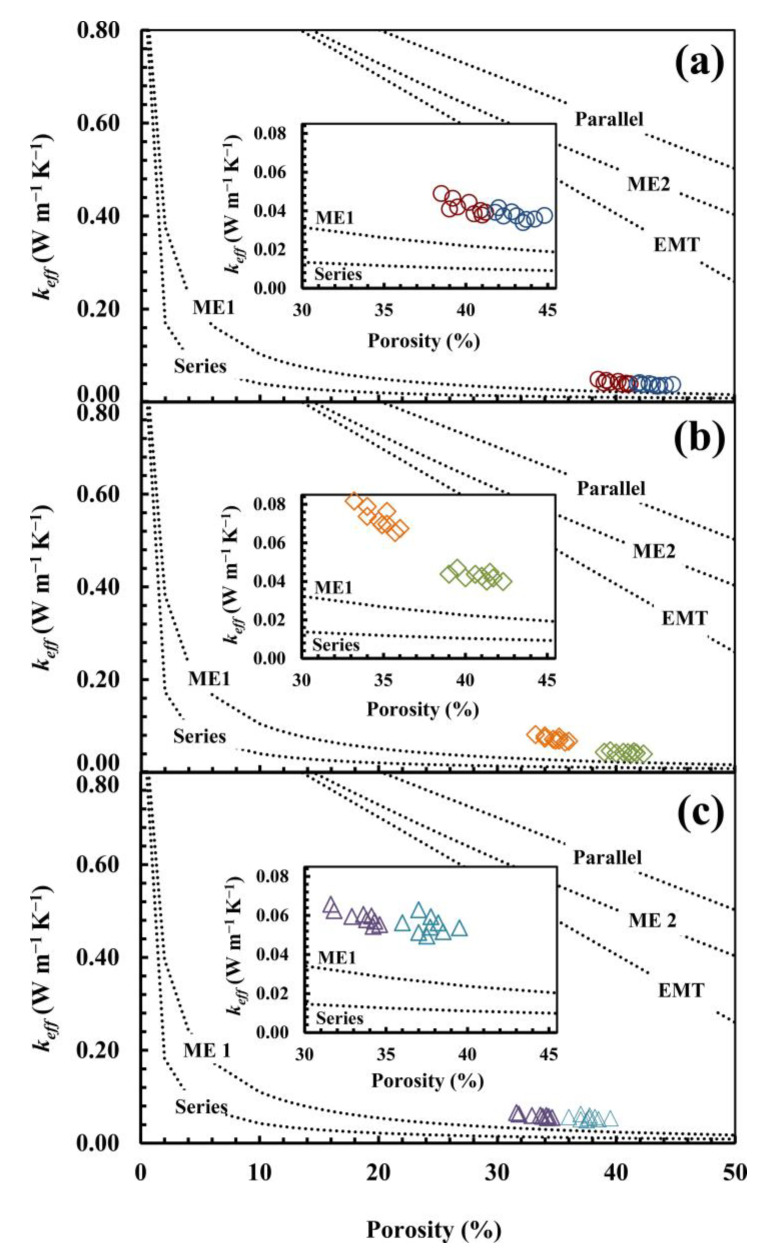
Comparison between experimental effective thermal conductivity (*k_eff_* = *k_exp_/k_s_*)—R-30 (**○**), R-40 (**○**), S-30 (**◊**), S-40 (**◊**), D-30 (**△**), and D-40 (**△**)—and model predictions (*k_eff_* = *k_mod_/k_s_*) using Parallel Equation (1), Series Equation (2), Maxwell–Eucken Equations (3) and (4), and EMT Equation (5). The calculation was performed for three different values of *k_s_*: 6.35 (**a**), 6.21 (**b**), and 5.86 W/m·K (**c**). *k_s_* was determined as a weighted arithmetic mean by considering the mineralogical composition thermal conductivity.

In order to better understand the thermal behaviour of the studied materials, traditional models and their relative analytical formulas (Equations (1)–(5)) were applied. The results are reported in Figure 3, in which *k_eff_* (*k_eff_* = *k_mod_*/*k_s_; k_eff_* = *k_exp_*/*k_s_*) is expressed as a function of porosity percentage. *k_s_* is obtained as a weighted arithmetic mean by considering the thermal conductivity of the different materials that comprise the mix-design: 6.35 W/m·K, 6.21 W/m·K, and 5.86 W/m·K for R, S, and D, respectively. In parallel, the ME2 and EMT models predict values far from the experimental data. Although series and, in particular, ME1 models seem to be capable of replicating the experimental trends, the enlargements in Figure 3 show remarkable differences, especially for series S. This is probably due to the fact that these models are proposed for materials which have different structures and features.

**Figure 4:**
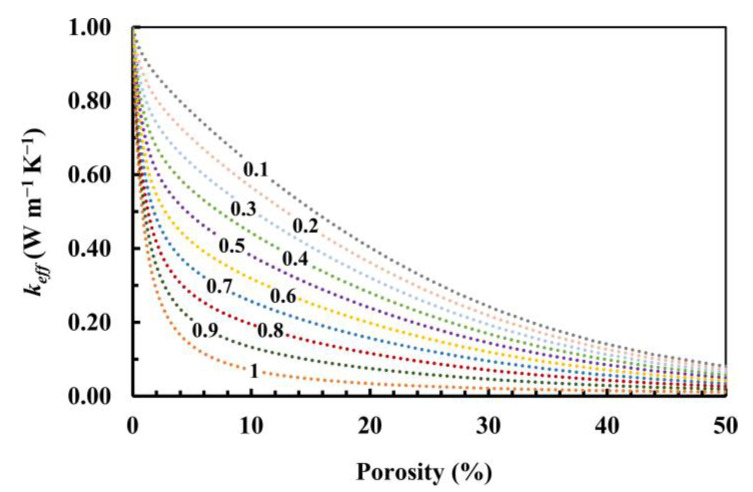
Parametric calculations of effective thermal conductivity vs. porosity considering *k_s_* = 6.35 W/m·K and varying *A_nt_*/*A* from 0.1 to 1.

For these reasons, a fractal model was proposed by Ma et al. (Equations (6) and (7)), which is formalized for granular materials such as those studied in this work. A parametric elaboration is reported in Figure 4, which was constructed considering *k_s_* = 6.35 W/m·K. By varying the *A_nt_*/*A* values, it is possible to obtain different curves, which highlights the great versatility of this model and its capabilities in giving relevant information on the material structure. Indeed, for the same porosity, the thermal conductivity has different values if referred to diverse *A_nt_*/*A* values. When the *A_nt_*/*A* decreases, the thermal conductivity increases. This trend is due to the topological organization of the structure. When the *A_nt_*/*A* decreases, the contact points between particles increases and the thermal resistance decreases. This could be related to the pore size distribution, in which, for the same porosity, a higher presence of smaller pores increases the non-touching area and consequently decreases the thermal conductivity [89].

**Figure 5:**
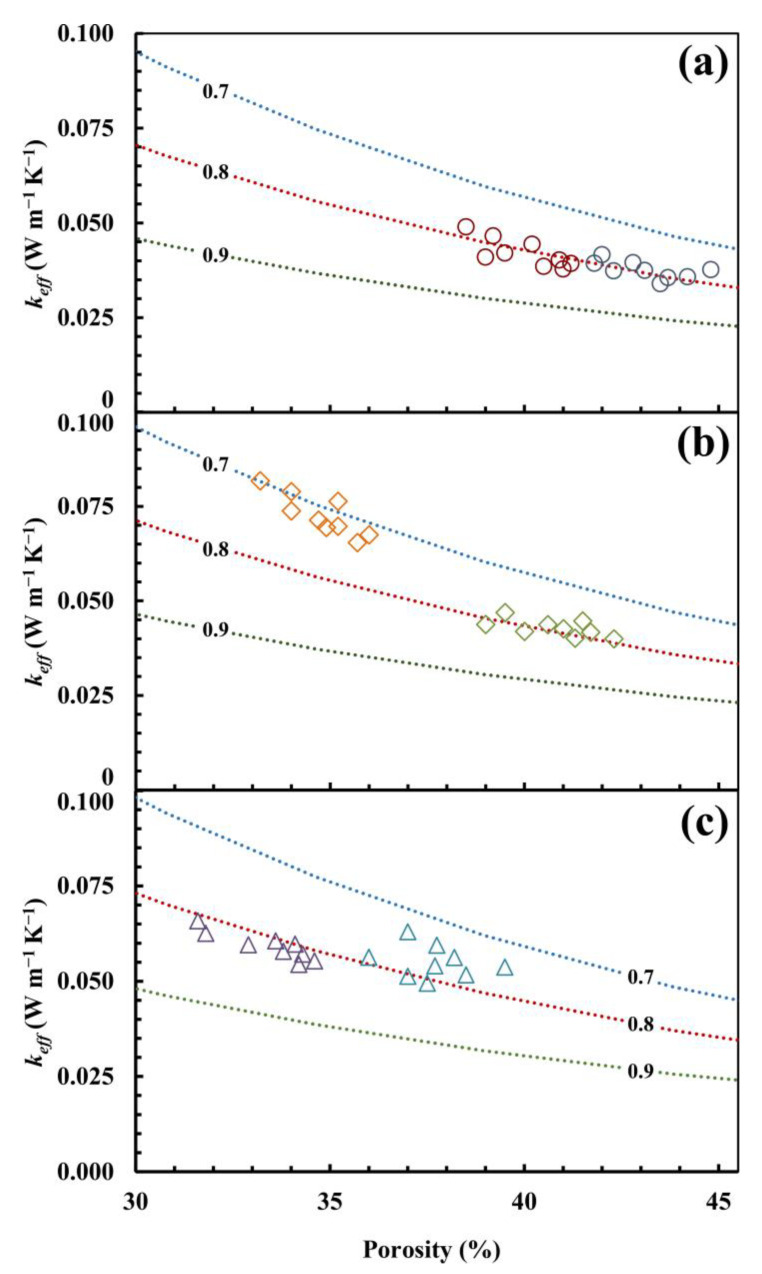
Comparison between experimental effective thermal conductivity (*k_eff_* = *k_exp_*/*k_s_*)—R-30 (**○**), R-40 (**○**), S-30 (**◊**), S-40 (**◊**), D-30 (**△**), D-40 (**△**)—and model predictions (*k_eff_* = *k_mod_*/*k_s_*) using Ma et al. The calculation was performed for three different values of *k_s_* 6.35 (**a**), 6.21 (**b**), and 5.86 W/m·K (**c**) and for the three different values of *A_nt_*/*A* reported in each panel: 0.7; 0.8; and 0.9. *k_s_* is determined as a weighted arithmetic mean by considering the mineralogical composition thermal conductivity.

Figure 5a–c shows the calculations carried out by using the Ma et al. model in order to fit the experimental data of R (*k_s_* = 6.35 W/m · K), S (*k_s_* = 6.21 W/m·K), and D (*k_s_* = 5.86 W/m·K), respectively.

It can clearly be noted that this model is able to fit the experimental data. For series R (R30, R40) and D (D30, D40), the best fit is obtained by using *A_nt_/A* = 0.8. However, the porosity range is quite different between the two systems. The presence of a large quantity of bio-based polymer influences the morphology of structure and generates a less porous system (D). Series S presents a difference between the average porosity of S30 and S40, respectively, equal to 35% and 41%. The relevant consideration in comparing S30 to S40 is an increase in the thermal conductivity values (S30 has a greater thermal conductivity than S40), which can be reproduced by using a different *A_nt_/A* (S30: *A_nt_/A* = 0.7 and S40: *A_nt_/A* = 0.8). This is due to the different quantity of water (water content: S30 25%, S40 35%) which, during the drying process, allows the particles to move closer, creating touching points. This condition is more conductive than the one that occurs in system D (particularly when comparing D30 to S30, systems with similar porosity), in which the greater quantity of bio-based polymer (20%) can reduce *A_nt_/A*.

Overall, the good agreement between the experimental data and modeling calculations indicates that the analytical model considered in this work is capable of suitably accounting for the thermal behaviour of earth-based materials, thus confirming the importance of considering the microstructure morphology of specimens, which can play a crucial role in heat conduction.

## 5. Conclusions

In this work, earth-based materials were characterized to assess their thermal behaviour in comparison to non-stabilized systems (as-received). Six different mix-designs were prepared: R30, R40 (with water contents of 30% and 40%, respectively), S30, S40 (containing a bio-based short oil alkyd emulsion at 5% and water contents of 25% and 35%, respectively), D30, and D40 (incorporating a bio-based urethane–alkyd dispersion at 20% and water contents of 10% and 20%, respectively).

The experimental measurements determined the mineralogical and granulometric composition of the raw materials constituting the earth used, as well as the density, porosity, and thermal conductivity of the fabricated systems after the mixing and drying phases.

As expected, an inverse relationship between the porosity and thermal conductivity was found. This phenomenon is more pronounced in the R and S series than in the D series, highlighting the influence of porosity on thermal transfer within these materials.

The general thermal behaviour was also investigated using traditional thermal models (series, parallel, Hashin and Shtrikman bounds, and EMT) and the subsequent fractal model by Ma et al. While traditional models appear incapable of reproducing the experimental data, the fractal model, elaborated for granular materials, shows remarkable versatility. Indeed, the model’s predictions closely align with the experimental data, allowing for the interpretation of results as a function of the microstructure. This approach poses a challenge for the construction of tools capable of suggesting mix-design solutions that lead to the preparation of samples with specific thermal behaviour. Moreover, further refinement could aid in understanding the relationships between the topology and morphology of microstructures and their macroscopic properties.

## Figures and Tables

**Table 1:** S-BAR and D-UAR bio-based polymer properties.

	S-BAR	D-UAR
Appearance	Liquid, slightly yellowish	Liquid, white, milky
pH	7.0–9.0	6.0–8.0
Bio-based content	>95%	>43%
Density	1.02–1.03 g/cm^3^	1.05 g/cm^3^

**Table 2:** The average density, the average porosity, and the average thermal conductivity of the systems and relative standard deviations.

Samples	Density (g/cm^3^)	Porosity (%)	*k_av_* (W/m·K)
R-30	1.586 ± 0.026	40.0 ± 1.0	0.267 ± 0.024
R-40	1.504 ± 0.027	43.1 ± 1.0	0.239 ± 0.015
S-30	1.683 ± 0.023	34.8 ± 0.9	0.451 ± 0.034
S-40	1.547 ± 0.029	40.8 ± 1.1	0.266 ± 0.014
D-30	1.690 ± 0.028	33.4 ± 1.1	0.347 ± 0.021
D-40	1.587 ± 0.030	37.7 ± 1.0	0.322 ± 0.025

## Data Availability

Data are contained within the article.

## Appendix A

The preliminary tests were designed to select mix-designs. Specifically, we sought the homogeneity of the sample in terms of surface uniformity, thus in the absence of cracking phenomena at the end of the drying process conducted in laboratory conditions. The studied mixes consist of CY and SD variables with a constant amount of distilled water for each series, set at 30% and 40%. The variation in the amount of water used relates to the minimum quantity necessary for the mixture to reach a consistency suitable for manual placement in the mold and the minimum amount of liquid phase usable for the mixture to be poured into the mold.

In particular, samples were made with the following quantities: 52% CY, 48% SD; 48% CY, 52% SD; 44% CY, 56% SD; 40% CY, 60% SD; and 36% CY, 64% SD. Only the last mixture showed no cracking and was therefore chosen as the starting mix-design for creating the samples.

The liquid phase in the mix-design was also kept constant (30 and 40%, respectively) as a result of introducing bio-sourced polymer to stabilize the material. The quantity of polymer was also chosen by seeking the sample’s homogeneity in terms of surface uniformity, thus in the absence of cracking phenomena following drying in laboratory conditions. S-BAR was tested at 3%, 5%, and 7%; D-UAR was tested at 3%, 5%, 7%, 15%, 20%, and 25%. From the visual analysis of the surfaces, it was determined that quantities above 5% of S-BAR and 20% of D-UAR caused surface cracking.
